# Genomics of fetal haemoglobin: a targeted approach for reticulocyte transcriptome study

**DOI:** 10.21203/rs.3.rs-3061395/v1

**Published:** 2023-06-30

**Authors:** Siana Nkya, Frida Kaywanga, Collin Nzunda, Salmaan Karim, David Solomon, Emmanuel Saukiwa, Heavenlight Christopher, Doreen Ngowi, Julieth Johansen, Florence Urio, Josephine Mgaya, Clara Chamba, Fadya Hashim, Emmanuela Ambroise, Solomon Ofori Acquah, Julie Makani

**Affiliations:** Muhimbili University of Health and Allied Sciences; Muhimbili University of Health and Allied Sciences; Muhimbili University of Health and Allied Sciences; Muhimbili University of Health and Allied Sciences; Muhimbili University of Health and Allied Sciences; Muhimbili University of Health and Allied Sciences; Muhimbili University of Health and Allied Sciences; Muhimbili University of Health and Allied Sciences; Muhimbili University of Health and Allied Sciences; Muhimbili University of Health and Allied Sciences; Muhimbili University of Health and Allied Sciences; Muhimbili University of Health and Allied Sciences; Mnazi mmoja Hospital; Catholic University of Health and Allied Sciences; University of Pittsburgh; Muhimbili University of Health and Allied Sciences

**Keywords:** Fetal haemoglobin, Reticulocyte enrichment, Immunomagnetic selection, RNA extraction, RNA integrity

## Abstract

**Background:**

Fetal haemoglobin (HbF) remains a major sickle cell disease modifier. The mechanism of HbF synthesis has been studied for several decades with the intention of increasing interventions for sickle cell disease (SCD), including drugs. However, the complex mechanism of HbF synthesis is influenced by multiple genetic factors interacting with environmental factors. In order to capture useful genetic information, especially with limited resources, one has to carefully design the study. This includes choosing the relevant participants, the correct phenotyping, the choice of samples, and the right genomic assays. This paper describes the approach undertaken as part of preparations for a reticulocyte transcriptome study intended to discover genes associated with HbF decline in newborns in Tanzania.

**Results:**

Of the 152 newborns enrolled in the larger study, 40 babies were selected for the reticulocyte transcriptome study based on their HbF levels at birth and later stage of life. Of these, 30 individuals were included under the category of high HbF levels ranging from 72.6–90% and the remaining 10 under the category of low HbF levels ranging from 5.9 – 10.3%. The reticulocyte enrichment recovery purity ranged from 85% - 97%. The total RNA concentrations obtained were >250 ng total RNA, with the average purity of 1.9 (A 260/280) respectively. The total concentration obtained was sufficient for the transcriptome and other downstream assays.

**Conclusion:**

We have documented important steps and factors to consider in identifying the relevant participants and required laboratory sample processes prior to the final stage, which involves total reticulocyte RNA sequencing.

## Background

Interventions for lifetime conditions such as sickle cell disease (SCD) will be more beneficial if tailored in a personalized manner. Fetal haemoglobin (HbF) is a known major SCD modifier and therefore a target for interventional developments, including drugs (1–3). The natural phenomenon of HbF synthesis has been studied as one approach to discovering interventions for SCD (4). A complex mechanism underlies the synthesis of HbF, which is a quantitative trait influenced by multiple genes and environmental factors (5–8).

Understanding the differential gene expression during HbF decline is expected to contribute to the search for potential drug targets as well as gene therapy for SCD. One of the approaches has been to investigate genes that are switched on and off in different cell populations at different stages of erythropoiesis (6, 9). Since the switching of fetal haemoglobin to adult haemoglobin starts before birth, it would have been ideal to study this phenomenon as the foetus is developing, particularly just before birth. However, this is not straightforward and brings immense complications. Another approach is to study the reticulocytes as they form a cell population during the final stage of erythroblast maturation. Reticulocytes are also the latest erythroid cell population with remnant genetic materials, which include those associated with the regulation and synthesis of haemoglobin, including HbF (10). The aim here would be to investigate the differential gene expression in reticulocytes. Our study involved investigating reticulocytes enriched from cord and peripheral blood.

In order for such a study to generate relevant data and due to cost limitations for such genomic studies, it is important to conduct a careful selection of participants, preferably those with extreme HbF levels. Studying individuals with extreme phenotypes (EP) enhances the identification of genetic variants that are strongly associated with a particular trait or disease, in this case HbF.(11) Such variants may be easily missed when studying individuals with more moderate or common phenotypes. Studying extreme cases helps pinpoint specific genes or regions of the genome that play a crucial role in determining the regulation of HbF. Similarly, individuals with extreme phenotypes are mostly associated with either milder or more severe diseases. Therefore, studying such individuals is helpful in understanding disease mechanisms as well as identifying potential drug targets. In addition, studying individuals with extreme phenotypes enhances statistical power. Our approach leads to an increased effect size of the genetic variants being investigated. This is especially important for complex traits or illnesses where small effect sizes for individual variants may exist. By concentrating on extreme cases, scientists can discover genetic variations that have a greater influence on the phenotype, producing more accurate and reliable results. This study outlines the procedures that are necessary to improve the accuracy of RNAseq and analysis for examining the reticulocyte transcriptomes in newborns with high HbF levels.

## Results

### Identification of study participants:

**Figure 1.**
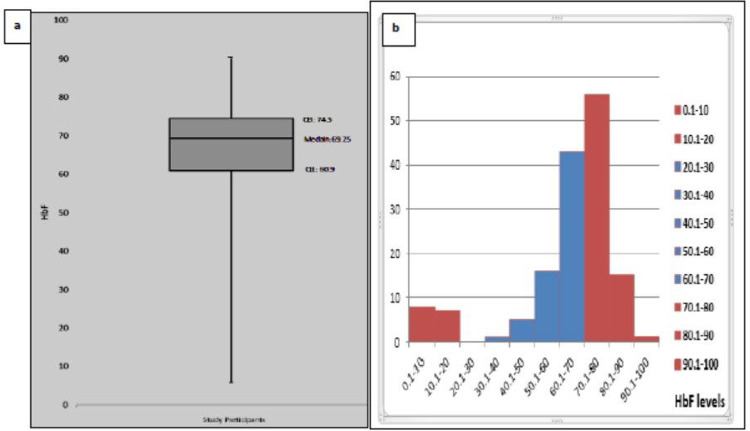
a) A box plot representation of the recruited individuals n=152. The calculated high interquartile range > (Q:74.5), indicating extreme high HbF individuals. b) Levels of HbF of screened individuals, n=152, mean = 63.4 range 5.9 – 90.2.

A total of 152 newborns were screened for the reticulocyte transcriptome study from December 2019 to November 2022. A total of 40 individuals were found to fit under the extreme category, 30 with extreme high HbF (>74.5, ranging from 72.6–90.2) and 10 with extreme low HbF (< 21.65, ranging from .9 – 10.3) (Figure 1).

### Enrichment of reticulocytes:

**Figure 2.**
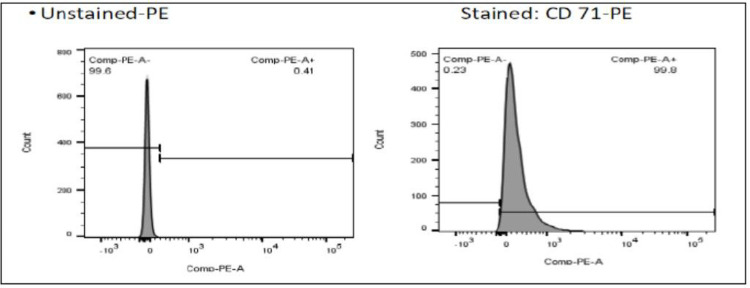
Reticulocyte recovery purity 99.8 after staining with CD71-PE antibody and analysis with FlowJo. Unstained sample (unexposed to any fluorescent reagent) was used as control.

A recovery purity of >85% was achieved following the adopted enrichment assay. This purity was mainly achieved through leukofiltration by Ficoll and Percoll centrifugation media coupled with negative selection by CD45+. A flow cytometry analysis was used to verify the purity (Figure 2).

#### RNA extraction:

Recovered concentration of RNA upon measurement by qubit fluorimeter 3.0 was >250 ng total RNA for all the samples and the purity of RNA as measured by Nanodrop at the absorbance ratio of 260/280 was average of 1.96.

## Discussion

Identifying genetic factors associated with fetal haemoglobin remains an important gateway to increasing alternative interventions for the management and treatment of SCD. However, identifying the right genetic factors requires a robust design, taking into consideration many factors that are not always straightforward. This is more difficult in low and middle - income countries where resources are limited. This paper describes the approach undertaken in Tanzania as part of an ongoing study aimed at studying the reticulocyte transcriptome in newborns with high and low levels of fetal haemoglobin. We focused our transcriptome study on selected newborns at birth and at a later stage, which provided us with those with extreme HbF levels (very high/low HbF). This approach is scientifically backed up and proven to enhance the identification of genetic variants, study disease mechanisms, enhance statistical power, contribute to personalized medicine, and study genetic architecture.

The selection of participants was based on the distribution of HbF levels established in our population. The individuals included in the extreme high HbF category were those above the upper quartile. Our study design, which involved measuring HbF at birth (using cord blood), provided us with a good selection of individuals with very high HbF levels (72.6–90.2). This is true because HbF is at the highest level at this point in any individual’s life. For the participants with low HbF (5.9–10.3) we selected babies at 18 months, at which HbF has declined to a significant level. Our original plan was to include both babies with very high and low HbF at birth. However, it was challenging as most babies had very high levels of HbF. Studies with a larger sample size that can allow for this design may benefit from studying and comparing the gene expression profiles of babies with extreme HbF levels at birth.

Our approach prioritized studying reticulocyte transcriptomes over other erythroid cell populations. This is because they are a cell population synthesized at the final stages of erythroid differentiation. Reticulocytes are also the only cell population with remnant genetic material with robust descriptions of genes, including those being highly expressed. However, reticulocytes are just a small proportion of cells found in either the cord (3.75 ± 1.16% with a range of 1.41–8.4%) or peripheral blood (2–6%). Therefore, in order to get sufficient cells for the transcriptome, the reticulocytes should be enriched and purified. We adopted the reticulocyte enrichment method by Skulski et al., 2019 (12). Enrichment of reticulocytes and flow cytometry analysis showed good recovery ranging from 85–97% in the category of individuals with extremely high HbF, while those with extremely low HbF had recovery purity of 32.4–48% respectively. The higher recovery of reticulocytes in the extreme high category than the low category is theoretically supported considering the fact that cord blood (the sample used for the high HbF category) contains a higher percentage of reticulocytes (4–5%) than peripheral blood (the sample used for the low HbF category)(13). However, for both categories, the recovered reticulocytes were sufficient for the isolation of RNA.

Concentrations of RNA recovered from both high and low categories ranged from 280–1500 ng total RNA, which is sufficient for the transcriptome study. Due to the sensitivity of the RNA sequencing assay it was necessary to ensure that the isolated RNA was of good quality. This was achieved using analysis by Qubit and through RNA integrity check using gel electrophoresis. On average RNA purity for our samples was sufficient for the transcriptome study, however better quality could be achieved by utilising more efficient RNA isolation systems and increasing the starting number of reticulocytes. Similarly, RNA integrity and intact assessment by gel electrophoresis indicated a non- degraded RNA with no contamination of gDNA as shown in Fig. 4

## Conclusion

We have provided a detailed account of the targeted approach for implementing a reticulocyte transcriptome study in our population. Our expectations are, this study will contribute significantly to the genomics of HbF especially in identifying potential targets for interventions which have a great impact for individuals with SCD.

## Methods

### Study sites and study population

The study was conducted at Mbagala and Sinza hospitals after obtaining ethical clearance from the Muhimbili University Institutional Ethics Review Board (IRB). The study involved newborns and infants (below the age of 18 months). Informed consent was obtained prior to enrolment into the study.

### Sample collection

Cord blood collection was conducted by well trained nurses following the standard cord blood collection protocol(14). Immediately after birth, the umbilical cord was clamped at two ends, with the distance between them being 8 to 10 inches. The section between the clamps was cut, and a blood sample (4.5 ml) was collected. Samples were transferred to the Hematology Clinical and Research Laboratory - MUHAS for processing. For infants, 2 ml of peripheral blood was collected in 5 ml EDTA tubes.

#### Quantification of HbF levels and categorisation of individuals with extreme levels

**Figure 3.**
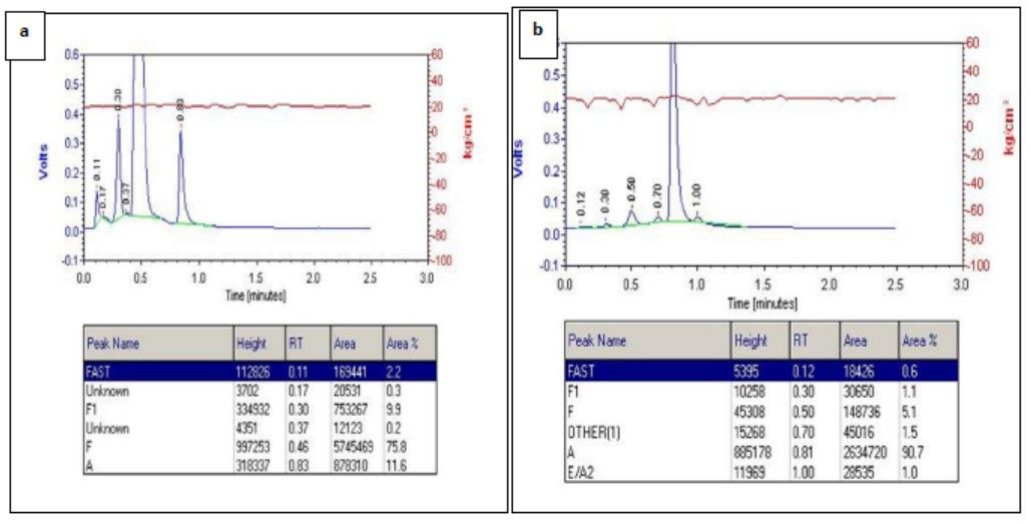
a) High-performance liquid chromatography output from the Bio-Rad VNBS instrument (Bio-Rad Laboratories, Hercules, United States) showing extreme high HbF of 85.7 total (sample from a newborn). b) High-performance liquid chromatography output from the Bio-Rad VNBS instrument (Bio-Rad Laboratories, Hercules, United States) showing extreme low HbF 6.2 total (sample from a baby with 18 months).

Fetal haemoglobin quantification was determined by using variant high performance liquid chromatography (VNBS-HPLC) (Bio-Rad Laboratories, Inc., USA). Briefly, samples were mixed properly in a Stuart rollermixer SRT6D for 5 minutes at a speed of 35 revolutions per minute (RPM). In a 96 well plate, 5ul of blood was added in a well followed with 250 ul of distilled water and eluted for 30 minutes in a Heidolph Titramax shaker, which was then run in a VNBS Biorad HPLC machine (Figs. 3). The extreme HbF levels were established following the upper and lower interquartile ranges, which were calculated from the HbF levels of the total population (152 individuals).

#### Reticulocyte enrichment

This procedure was conducted to enrich reticulocytes from blood samples collected from the identified individuals with extreme HbF levels. The procedure of enrichment included (4) main steps. i.e *Leukofiltration, Washing of beads, Negative selection with CD45 + cells, and Enrichment of CD71 + cells* as adopted from previously reported methods (12). Leukofiltration, was attained by using ficoll paque plus and Hanks balanced salt solution (HBSS) and was aimed at removing mononuclear cells. Washing of beads involved washing of CD45 + and CD71 + beads that were used for negative and positive selection i.e selection/depletion of leukocytes and enrichment of reticulocytes, respectively.

**Flow Cytometry** was done by FACS Canto BD to analyze the recovered purity of the CD71 + enriched cells i.e reticulocytes. The antibody/stain ratio of 1:1 of 10 ul each was used, and cells were incubated for 20 minutes in a dark place at RT and washed twice with PBS before proceeding for flow cytometry. Cells with high recovery purity of about 85%- 97% proceeded for RNA extraction.

#### RNA isolation

The RNA extraction procedure was done by using Invitrogen Pure Link RNA mini kit (Cat no. 12183018A) from Thermo Scientific U.S.A. A total of five (5) steps were employed in the procedure, which were, lysis and homogenization, binding purification, washing, and elution. The lysing of samples was performed by using 2-beta mecaptomercaptol ethanol and lysis buffer according to the manufacturer’s protocol. Manual homogenization was performed for 5 minutes and centrifuged for 2min by 12,000g at 4°C. 70% of the ethanol was added to the cell homogenate and passed in the spin cartridge for binding with 12000 G centrifugation at RT for 15 seconds. As per manufacturer’s protocol, washing was done by using buffer I and II with the centrifugal force of 12000 G for 15 seconds. RNase free water was used for elution. 50ul of RNAse free water was added. Incubation was done for one minute and centrifugation (12000g) to recover RNA.

##### RNA purification, quantification, and purity check:

After extraction, RNA was purified with a (1:1) RNA-DNase ratio and incubated for 15 minutes at 37°C, thereafter passed in a spin column to remove any gDNA contamination. Two instruments were used for determining the quantity and purity of isolated RNA, namely the Qubit Fluorometer 3 and NanoDrop spectrophotometer, respectively. During measurement by Nanodrop, RNase free water was used as the blank solution before each sample measurement. 2μL of RNA were placed on the pedestal, the lid was closed, and the RNA concentration was measured. Measurement by Qubit Fluorometer 3.0 was done using the Qubit^™^ RNA High Sensitivity (HS) assay kit. Prior to every run, the Qubit 3.0 fluorometer was calibrated using the standards provided in the kit, and each run control was used.

##### RNA integrity analysis:

**Figure 4.**
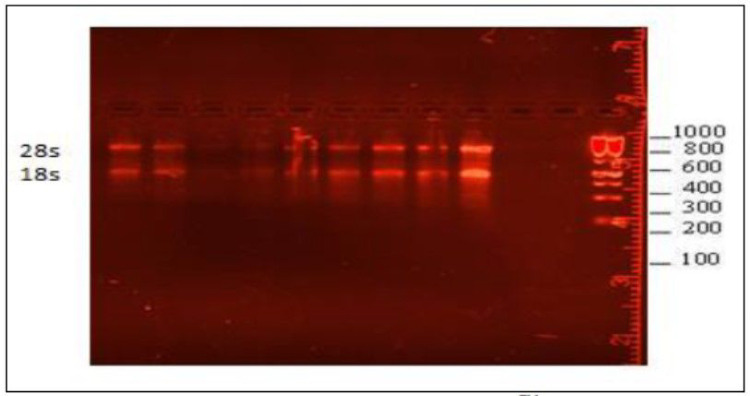
Gel image, visualized under Molecular Imager Gel Doc^™^ XR+ showing clear 28s and 18s RNA bands after degradation of gDNA with DNase addition.

The gel electrophoresis method was used to check RNA integrity. RNA with a known concentration was run through a gel electrophoresis in order to visualize RNA intactness, size, and overall integrity. Agarose gel of 0.8% was used for the integrity analysis. Molecular Imager Gel Doc^™^ XR + with Image was used for visualization using lab software from Bio-Rad Laboratories Inc, USA (Fig. 4). Upon waiting for scheduled transcriptomics assays, RNA was stored at room temperature after addition of RNA shield (1:1).

## Data Availability

The datasets used and/or analysed during the current study are available from the corresponding author on reasonable request.

## Abbreviations

Amp: Ampere
CD45: Cluster of differentiation 45
CD71: Cluster of differentiation 71
DRC: Democratic Republic of Congo
EDTA: EthylAmineTetraaceticacid
EP: Extreme phenotypes
gDNA: Genomic DNA
HbL: Haemoglobin
HbF: Fetal haemoglobin
HbA: Adult Haemoglobin
HS: High Sensitivity
HPLC: High Performance Liquid Chromatography
IRB: Institutional Review Board
MUHAS: Muhimbili University of Health and Allied Sciences
NCD: Non-Communicable diseases
Ng: Nanogram
RCF: Revolution Centrifugal Force
RNA: Reboxy nucleic acid
RIN: RNA Integrity Number
RPM: Revolution per minute
RT: Room Temperature
SCD: Sickle Cell Disease
TBE: Tris Borate EDTA
V: Voltage
